# Comparative Study of Hops Moisture Content and the Relative Humidity of the Drying Environment in a Hop Belt Dryer

**DOI:** 10.3390/s25154526

**Published:** 2025-07-22

**Authors:** Petr Heřmánek, Adolf Rybka, Ivo Honzík, Tomáš Hlavsa, Jiří Marčan

**Affiliations:** 1 Faculty of Engineering, Department of Agricultural Machines, Czech University of Life Sciences Prague, Kamýcká 129, CZ165 00 Praha, Czech Republic; rybka@tf.czu.cz (A.R.); honzik@tf.czu.cz (I.H.); marcan@tf.czu.cz (J.M.); 2 Faculty of Economics and Management, Department of Statistics, Czech University of Life Sciences Prague, Kamýcká 129, CZ165 00 Praha, Czech Republic; hlavsa@pef.czu.cz

**Keywords:** hop, drying, belt dryer, relative humidity, moisture

## Abstract

The paper concerns a study of drying and the creation of a statistical model for measuring the relative humidity of the drying environment in a belt dryer, as well as the moisture content of hop heads, stems, and bracts. The SAAZ variety was used, which is widely cultivated in the Czech Republic, and the data from harvesting seasons since 2017 were recorded. The findings demonstrated the influence and dependence of the moisture content of hop cones and their parts on the relative humidity of the drying environment in a belt dryer of hops. This dependence was confirmed by a statistical analysis of the measured values. Furthermore, a quadratic model was developed based on measurements taken over three harvest seasons. The model is applicable to predict the moisture content of the hops at a given location based on the relative humidity of the drying environment in the belt dryer and could be useful for developing an automatic hop-drying system.

## 1. Introduction

Hops (*Humulus lupulus* L.) has been traditionally used for its sedative, antioxidant, and antimicrobial properties [1], as well as for culinary purposes. More than 90% of hops produced worldwide is used as a bittering agent in brewing; additionally, it also adds aroma to beer [2]. Nevertheless, the biological characteristics of hops suggest its potential usage in therapeutic and nutraceutical applications [3,4,5,6].

Humulones (α-bitter acids) in soft resins of the hops are the most important constituents that determine the quality of hops. Several studies have demonstrated that pre- and post-harvest factors, as well as maturation time, can affect the humulone concentration in hops. Another important parameter is the content of essential oils and their mutual ratio [7,8,9].

Drying is an ancient method used for preserving agricultural materials and food. Hot and natural air drying are commonly used to dry medicinal and aromatic plants; however, these methods have disadvantages—natural air drying takes a long time, while hot air drying reduces the quality of the materials. In contrast, microwave drying drastically reduces drying time; however, it causes significant oil loss and discolouration [10]. Brushed cones have a moisture of 70–90% [11]. Following harvest, hop cones exhibit high metabolic activity, particularly aerobic cellular respiration, which leads to the oxidation of stored carbohydrates and results in the release of carbon dioxide, water, and thermal energy. The accumulation of this metabolic heat within the moist cone tissues can raise their internal temperature, thereby increasing the risk of self-steaming and subsequent quality degradation [12]. Therefore, hops require immediate preservation after being brushed to prevent deterioration. Convective drying is the most widely used method for hops preservation. It is a drying process in which the drying medium (usually air), which is also a heat-carrying medium, passes through the dried hops and simultaneously exchanges heat and mass. Most growers worldwide dry hops at 55–60 °C for 6–8 h in chamber louvre or belt dryers on 10–12% final moisture [13,14]. Based on growers’ experience, it can be stated that these temperatures and drying times are relatively suitable for the finalization of hops products for brewing technology. However, the mentioned temperature range, mainly in the final drying phase, and its long duration hinder the preservation of key hops [15]. Furthermore, simultaneous use of the drying parameters may lead to irreversible transformations and losses. In other words, these factors negatively affect the subsequent use of hops in medicines, food supplements, and cosmetics.

A successful drying process involves the fastest elimination of water from materials and food while preserving their quality and minimizing energy consumption using a dryer. The success and sustainability of drying are achieved by maintaining the quality of the dried products, measurable energy consumption, the ability to dry various wet materials, and investment/operating costs [16,17,18,19]. Furthermore, the use of alternative energy sources to operate the dryer, the reduction in dryer costs, and the use of drying equipment for different wet materials all play a role in the environmental and economic sustainability of the dryer. To overcome the financial and technological challenges encountered by the agriculture and related industries owing to the unique drying conditions required for different materials, over 400 dryers have been developed and used [10,19,20,21,22,23].

Several studies have developed different drying models depending on the materials [24,25,26,27,28]. Winiczenko et al. [29] investigated the effects of drying temperature and air velocity on various parameters, including water absorption capacity, volume ratio, and colour difference applying conventional drying. The researchers developed an optimization algorithm based on Pareto optimality, a genetic algorithm, and an artificial neural network. Kaveh et al. [30] predicted the convective drying parameters of various agricultural products using an adaptive neuro-fuzzy inference system and artificial neural networks. Zhang, Ma, and Yang (2015) [31] proposed a method for controlling the temperature and relative humidity separation in dryer control systems. The simulation results showed that the relative humidity fluctuations were significantly reduced. However, no study has reported the numerical models and networks during the drying of hops.

Growers use two main types of hop dryers for hops drying—belt dryers and chamber dryers. Belt dryers are the most common, accounting for approximately 60% of installations. Alternative drying systems, such as deep bed dryers (e.g., American Deep Bed Drying) or multi-deck kilns, could theoretically be used for hops; however, these systems are not well suited for drying such a specific plant material as hops. Historically, a counterflow drying principle has become standard in hops drying, in which the airflow is directed opposite to, or perpendicular to, the movement of the hops material through the dryer [32].

In comparison to belt dryers, chamber dryers offer only one significant advantage: substantially lower space requirements for the drying facility. However, their major drawback is the lack of continuity in the overall drying process.

Belt dryers became widespread in the second half of the 20th century. These dryers, typically consisting of three drying belts, offer continuous operation, higher throughput, the ability to increase the thickness of the hop layer, and suitability for integration into a complete harvest line. An important innovation is the possibility of mixing and turning hop layers as the material transfers between belts, which enhances moisture removal from the hops [33].

In the Czech Republic, hops are currently cultivated on an area of 4816 hectares, of which nearly 4500 hectares are planted with the SAAZ variety, a globally renowned cultivar. From this total area, approximately 6300 tons of hops are produced annually, with around 6000 tons represented by the SAAZ variety. This dominance is historically established, and it is expected to remain unchanged in the foreseeable future [34].

The primary objective of this study was to investigate the relationship between the residual moisture content in hop cones and their morphological components (bracts and strigs) and the relative humidity of the drying air in a belt dryer system. Relative humidity constitutes a critical parameter influencing drying kinetics and moisture diffusion behaviour in plant materials and is thus essential for the effective control and optimization of the hops drying process.

Understanding the moisture dynamics in specific anatomical structures of hops under varying drying conditions provides a scientific basis for the development of intelligent, automated control systems. Such systems are intended to enhance drying efficiency and product consistency. Given the current lack of non-invasive, real-time moisture measurement technologies applicable to hops, the findings of this research are particularly relevant for advancing precision agriculture technologies and supporting the implementation of data-driven, adaptive drying protocols in industrial hops processing.

## 2. Materials and Methods

**Figure 1:**
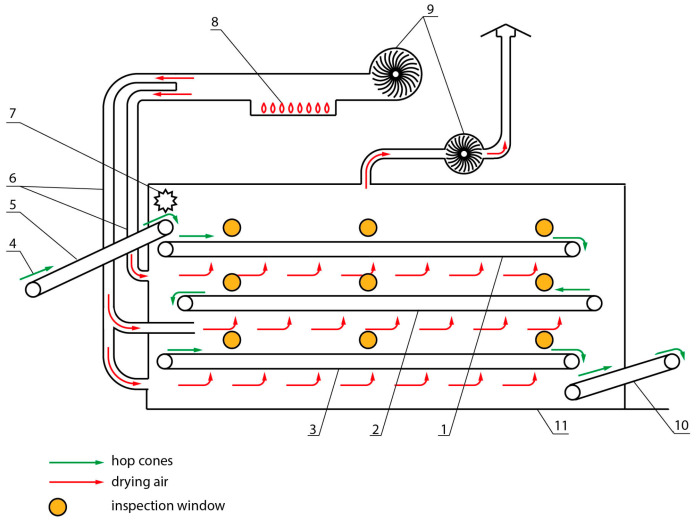
Scheme of the hop belt dryer PCHB 750. 1—upper drying belt conveyor, 2—middle drying belt conveyor, 3—lower drying belt conveyor, 4—wet hop cones (80% moisture), 5—inclined conveyor, 6—distribution air pipe, 7—straightening roller, 8—hot air unit, 9—fan, 10—belt of dry hops (10–12% moisture), 11—drying section.

In this paper, SAAZ was selected, i.e., the variety most widely used in the Czech Republic. Hops samples for measurement were taken from a PCHB 750 hop belt dryer (Figure 1) at Agrospol Velká Bystřice Co., Ltd. (Velká Bystřice, Czech Republic), located in the Olomouc Region in the Czech Republic. The measurement has been taking place since 2017.

The temperature and relative humidity of the drying environment and the moisture content of the hops were determined as follows:

- By measuring with embedded data loggers (DL 1–3) VOLTCRAFT DL-121-TH (Conrad Electronic);
- Measurement using Comet T3419 fixed sensors (FS) and the Comet MS6D measuring centre;
- Laboratory analysis (LAB) of samples obtained on a halogen moisture analyser HE43 from Mettler-Toledo.

Each of these methods requires specific measurement conditions. When observing the correlation between the moisture of hop cones and their components (bracts and strigs) and the relative humidity of the drying environment in a hop belt dryer, data are from inspection windows 1–9.

### 2.1. Measurements Using Data Loggers (DL 1–3)

VOLTCRAFT DL-121-TH data loggers were used to continuously measure the temperature and relative humidity of the air in the dried hop layer, and the frequency of data storage was programmed. In this study, the data-saving frequency was set to 5 min. The internal memory of the data logger could hold 32,000 measured data points, which was sufficient. The data logger was integrated with the sensor in a plastic case and was powered by an embedded battery. The plastic case was equipped with a USB connector at one end through which the saved data were imported to a computer. To protect against mechanical damage during passage through the dryer and contamination, the data loggers were fixed in polyurethane and inserted into two hemispherical stainless-steel sieves. This ensured sufficient protection, and at the same time, the sieves did not obstruct the passage of air, and thus, measurement errors did not occur.

During the drying process, data loggers were used to continuously record and monitor the entire process. These loggers were carefully placed into the belt dryers through the first inspection window, i.e., three pieces simultaneously. They were positioned about 1 m from both ends of the belt and in the middle. Once the samples were passed through the entire dryer, they were removed. Additionally, a battery-powered LED light was inserted into the dryer along with the data loggers. This light moved along the hop layer and signalled when the data logger reached the border of the inspection window. The average belt speed between the inspection windows was calculated using light malting. When the light reached the axis of the inspection window, the samples were collected for laboratory tests. This ensured that data from the fixed sensors, data loggers, and sampling were obtained simultaneously.

### 2.2. Measurements Using Fixed Sensors (FS)

**Figure 2:**
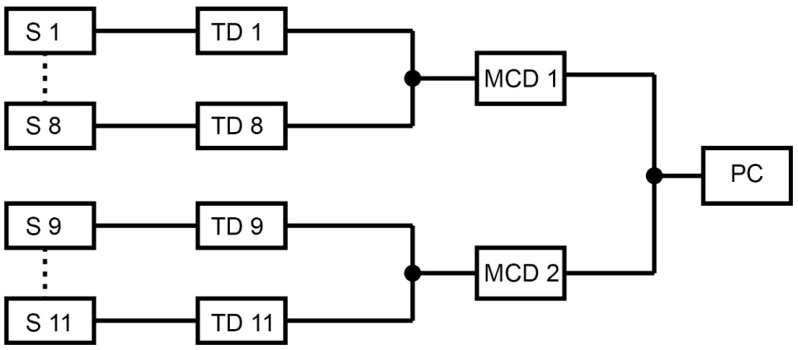
Connection diagram of sensors, measuring centres and the computer. S 1–S 11: Sensor, TD 1–TD 11: Transmitter and display, MCD 1–MCD 2: Multi-channel data logger, and PC: Computer.

The belt dryer was fitted with Comet T3419 fixed-temperature and relative-humidity sensors. Each set of eight sensors was connected to a Comet MS6D control panel. Installing 11 sensors and two measuring centres in one dryer was necessary. The switchboard data were automatically saved to a computer on a hard disk (Figure 2).

The reading frequency was set to 5 min, which is similar to that of the data loggers. Instantaneous measured values were determined from the attached two-line display, on which the instantaneous temperature in °C and relative air humidity in % were displayed simultaneously. Along with the data on temperature and relative humidity, the measurement time was also saved, with the help of which it was possible to match data from different measurement methods.

### 2.3. The Laboratory Analyses of the Acquired Samples (LAB)

**Figure 3:**
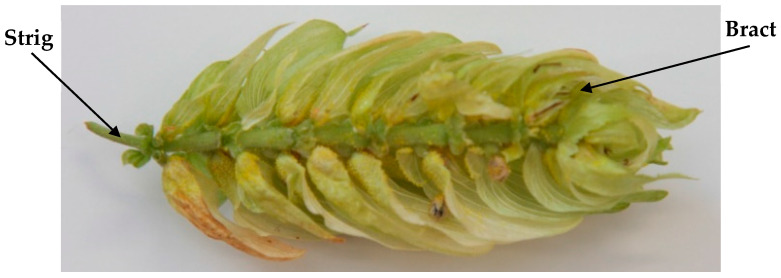
Representative image of a hop cone.

During the laboratory analyses, the moisture content of the collected hop, strigs, and bracts samples (Figure 3) was monitored and compared with the relative humidity of the drying medium measured using data loggers and fixed sensors on the dryers. The samples for laboratory testing were collected from each inspection window. The moisture content of the hops on the moisture analyser was also determined when the moisture content of the sample was continuously displayed on the device. The weight of the sample, in this case, was approximately 3 g. The measurement ended when the weight loss of the sample during the defined time interval was less than the pre-measured value. The HE43 halogen moisture analyser from the Mettler-Toledo company has proven to be suitable for measuring the moisture content of hops.

Modelling was also performed of the relationship between the relative humidity of the environment and hops cone moisture. A regression analysis was used to estimate the parameters of a parabolic function using the ordinary least squares method. The suitability of the model was assessed based on the coefficient of determination; the model, including its parameters, was tested using analysis of variance or the *t*-test. The residuals were also analysed, including a homoscedasticity test using White’s test and autocorrelation using the Durbin–Watson test [35,36]. The solution was analysed using the SAS Studio software version 9.4.

## 3. Results and Discussion

**Figure 4:**
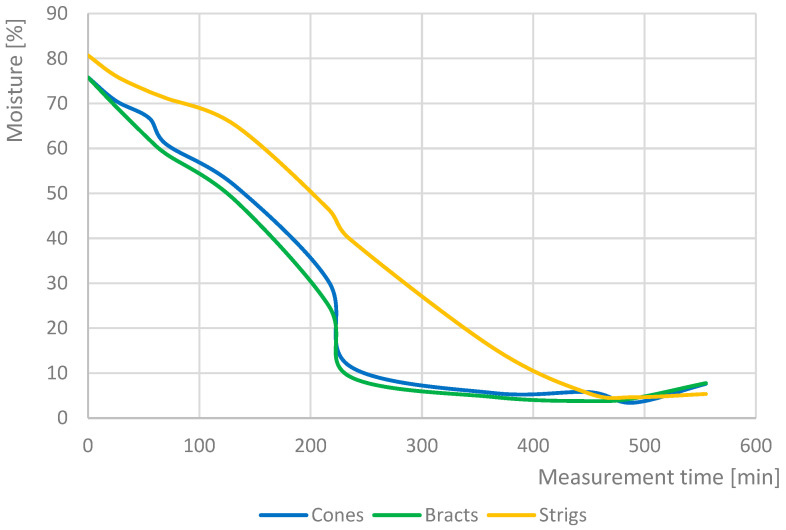
Dependence of moisture cones, bracts, and strigs on drying time (drying curve).

**Figure 5:**
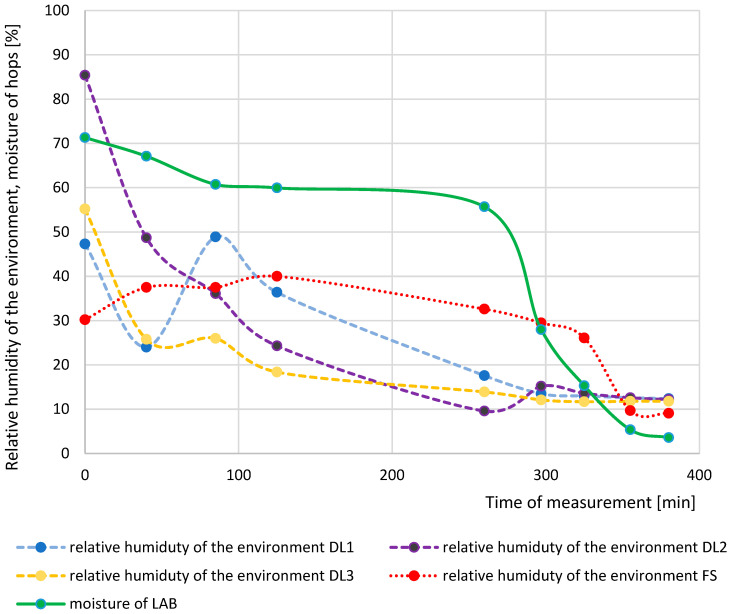
Dependence of the relative humidity of the drying environment and the moisture of hops on the measurement time in a belt dryer. DL 1, DL 2, and DL 3: measurement with data loggers, FS: measurement with fixed sensors, and LAB: laboratory measurement of hops moisture.

Depending on the measurement time, the hop cones and bracts have approximately the same moisture content (Figure 4). The drying trends for all the parts were similar. Concordant with this finding, Münsterer (2020) [37] showed a similar dependence of relative humidity on drying time. However, Figure 5 shows the drying process for the Czech hops variety SAAZ, and the results according to Münsterer (2020) [37] are for German hops varieties. Therefore, only the hop-cone moisture content was used in further analyses. Furthermore, as shown in Figure 4, the moisture of the strigs decreased very slowly compared to that of the bracts. Because the weight ratio between the bracts and strigs was approximately 8:2, measuring the moisture of the cones yields a value that is similar to that of the bracts. The data showed that the moisture of the cone was 12% at the exit of the second belt and that it was 30 to 40% in the strigs at the same time. Furthermore, upon pressing, the strigs release moisture, leading to the deterioration of hops. The release of moisture from hop cone strigs occurs only in cases of incomplete drying of the hop cones. Once the moisture content of the bracts and the strig equalizes, as shown in Figure 4 at 450 min, no further moisture transfer between the bracts and the strig takes place.

Figure 5 shows the relative humidity and moisture contents measured from three data loggers (DL 1–3), fixed sensors (FS), and hops moisture from laboratory analysis (LAB) over the time of measurement. The progress of the DL1 and DL3 data loggers distributed across the width of the belt is practically identical. However, a difference was observed in the third inspection window due to the transition to the next belt and the accumulation of material before falling onto the second belt on one side of the dryer. The data obtained from sensor DL2, positioned at the centre of the belt, demonstrated a decreasing trend comparable to that observed in sensor DL3, indicating similar dynamics of relative humidity in these regions of the drying layer. The relative humidity measurements in the drying environment using fixed sensors indicated a similar trend, albeit with minor variations. The results of the laboratory analyses of hop moisture corresponded to the relative humidity of the drying environment. Given the variability observed in the relative humidity measurements from sensors DL1–3, the modelling was based on data obtained from the fixed sensors (FS), which provided more stable and representative environmental readings.

**Figure 6:**
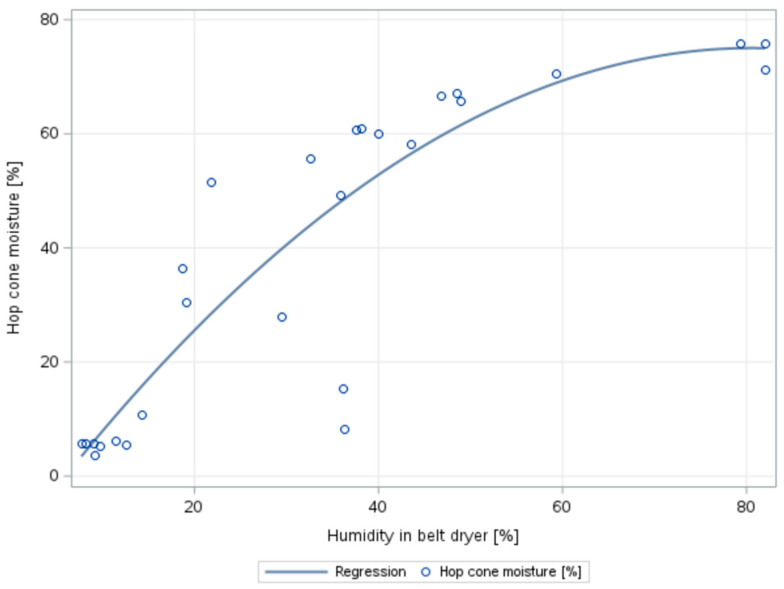
Course of the dependence of the moisture of the hop cones on the relative humidity of the drying environment based on data from three harvest seasons, self-processing in the software SAS studio.

Subsequently, statistical analyses were performed based on data from the three harvest seasons. The dependence of hop cone moisture on the relative humidity of the environment was also monitored. The purpose is to examine the relationship between these variables and to build a model that could be used for the prediction of the hop cone moisture based on the relative humidity. Regression analysis was used for modelling. As shown in Figure 6, the hop cone moisture decreased with a reduction in the relative humidity of the drying environment.

Sensors DL1 and DL3 were placed on the belt 0.5 m from the edge, while sensor DL2 was positioned in the centre of the belt, 1.5 m from the edge. This sensor arrangement was used to monitor the drying environment parameters across the full 3 m width of the hop layer. The relative humidity values recorded by sensors DL1 to DL3 were not consistent. The factors contributing to these differences include the uneven moisture content of the incoming hops, non-uniform distribution of hops on the belt, varying layer thickness, and uneven airflow of drying air through the hop layer.

However, this relationship is not linear but is better explained by a quadratic function. The model is estimated using the following parameters:

Ŷ = (−12.47) + 2.17X − 0.013X^2^ (1)

The quadratic model explained almost 80% of the variability in head humidity (R^2^ = 0.7850). It is also statistically significant as a whole (analysis of variance for the test of all model parameters gives the result F = 43.82 and *p*-value = 0.0001, and the null hypothesis of non-significance of the parameters is rejected). Based on the *t*-tests of the individual parameters, it can be concluded that the parameters are statistically significant at the 0.05 significance level (the test of parameter 2.17 gives the test statistic t = 5.22, *p*-value = 0.0001, the test of the parameter (−0.013) has the test statistic t = −2.86, *p*-value = 0.0087; in both cases, the *p*-value is less than the significance level of 0.05, and the null hypothesis of independence was rejected). The normality of the residuals was analyzed, including the homoscedasticity test using White’s test (χ^2^ = 5.9, *p*-value = 0.3158), so that the residuals have the same variance, which is required by the assumptions of the least squares method used for parameter estimation. In addition, the model does not exhibit autocorrelation of residuals, based on the outcome of the Durbin–Watson test (DW = 1.535, *p*-value = 0.078). The results of the statistical tests prove that it is possible to use the model to estimate hop cone moisture depending on the relative humidity of the drying environment.

Thus, this model can be considered adequate and sufficiently accurate. At the same time, it can be stated that the model is relatively robust, as it is based on measurements since 2017. Similar trends in the development of head cone moisture have been documented by Sturm et al. (2020) [38], and the hop cone moisture prediction model developed in this study provides results similar to those of the same study. Suppose the regression model developed in this study is applied to estimate hop cone moisture. In this case, it can be stated that at the end of the first belt of the hop dryer (3rd window), the average moisture of the hop cones will be approximately 56%; at the end of the second belt (6th window), it will be approximately 27%; and at the end of the third belt (9th window), it will be approximately 5%. According to the measured values of the actual moisture of the cones, it is possible to state that the hops are already considerably dried or over-dried at the exit from the belt dryer.

## 4. Conclusions

The present study demonstrates the dependence of the moisture of hop cones and their parts (bracts and strigs) on the relative humidity of the drying environment in the belt dryer of hops. The study suggests that only the moisture of the whole cones should be chosen because it was possible to determine the instability of the moisture of the bracts in relation to a similar course of moisture of the bracts and strigs. The dependence of the moisture of the hop cones on the relative humidity of the drying environment in the hop belt dryer was verified statistically using a quadratic model and was general for the given belt dryer. This model was based on measurements taken since 2017 and is applicable to predict the moisture content of the hops at a given location based on the relative humidity of the drying environment in the belt dryer. To increase the stability of the model and make it more robust, the model should be refined annually based on prevailing harvest conditions. According to the measured moisture levels of the cones, it can be concluded that the hops samples tested in this study were already significantly dried or possibly even over-dried upon exiting the belt dryer. This model could be useful for the development of an automatic hop-drying system.

According to the results, it can be stated that for determining the moisture content of hops, it is sufficient to monitor the moisture content of the heads. According to the diagram in Figure 1, it is also evident that the current belt dryer, which was used in the research, does not have a high-quality distribution of drying air. Therefore, it is problematic to set a constant air temperature for exact drying research. The authors’ further work will consist of innovating the distribution of drying air and the subsequent design of drying automation on the belt dryer of hops.

The proposed model is specifically calibrated for the PCHB750 belt dryer with three belts. The model was developed based on experimental data obtained during the drying of hops of the SAAZ variety within a temperature range of 55–60 °C. Future research will focus on extending the model to analyse the effect of temperature on moisture content, including other significant hops varieties such as Premiant, Sládek, and Agnus.

The authors are also aware that this is a field where there can be considerable dependence on natural conditions such as weather and relative humidity. Overall, these external influences can contribute to certain inaccuracies. However, using the data from several years, they can be eliminated.

## Data Availability

The research data are available from the corresponding author upon reasonable request.

## Nomenclature

Co., Ltd.	Company Limited
CZU	Czech University of Life Sciences Prague
DL	data logger
F	parameter of statistical method
LAB	laboratory analysis
LED	light-emitting diode
MCD	multi-channel data logger
NAZV	National Agricultural Research Agency
PC	personal computer
PCHB	type of belt dryer from the manufacturer Vzduchotechnika, Nové Město nad Váhom, Slovak Republic
FS	fixed sensor
*p*-value	parameter of statistical method
R	parameter of statistical method
S	sensor
SAAZ	traditional Czech hop variety
SAS studio version 9.4	statistical software
t	parameter of statistical method
TD	transmitter and display
*t*-test	type of statistical analysis
USB	universal serial bus, the system for connecting other pieces of equipment to a computer
Χ	parameter of statistical method
